# Physiologic cold shock of *Moraxella catarrhalis *affects the expression of genes involved in the iron acquisition, serum resistance and immune evasion

**DOI:** 10.1186/1471-2180-11-182

**Published:** 2011-08-12

**Authors:** Violeta Spaniol, Rolf Troller, André Schaller, Christoph Aebi

**Affiliations:** 1 Institute for Infectious Diseases, University of Bern, CH-3010 Bern, Switzerland; 2 Division of Human Molecular Genetics, University of Bern, Inselspital, CH-3010 Bern, Switzerland; 3 Department of Pediatrics, University of Bern, Inselspital, CH-3010 Bern, Switzerland

## Abstract

**Background:**

*Moraxella catarrhalis*, a major nasopharyngeal pathogen of the human respiratory tract, is exposed to rapid downshifts of environmental temperature when humans breathe cold air. It was previously shown that the prevalence of pharyngeal colonization and respiratory tract infections caused by *M. catarrhalis *are greatest in winter. The aim of this study was to investigate how *M. catarrhalis *uses the physiologic exposure to cold air to upregulate pivotal survival systems in the pharynx that may contribute to *M. catarrhalis *virulence.

**Results:**

A 26°C cold shock induces the expression of genes involved in transferrin and lactoferrin acquisition, and enhances binding of these proteins on the surface of *M. catarrhalis*. Exposure of *M. catarrhalis *to 26°C upregulates the expression of UspA2, a major outer membrane protein involved in serum resistance, leading to improved binding of vitronectin which neutralizes the lethal effect of human complement. In contrast, cold shock decreases the expression of Hemagglutinin, a major adhesin, which mediates B cell response, and reduces immunoglobulin D-binding on the surface of *M. catarrhalis*.

**Conclusion:**

Cold shock of *M. catarrhalis *induces the expression of genes involved in iron acquisition, serum resistance and immune evasion. Thus, cold shock at a physiologically relevant temperature of 26°C induces in *M. catarrhalis *a complex of adaptive mechanisms that enables the bacterium to target their host cellular receptors or soluble effectors and may contribute to enhanced growth, colonization and virulence.

## Background

*Moraxella catarrhalis *colonizes the mucosal surface of the human nasopharynx and is a major cause of acute otitis media in children and of exacerbations of chronic obstructive pulmonary disease in adults [1,2]. Clinical studies have revealed that the prevalence of pharyngeal colonization and respiratory tract infections caused by *M. catarrhalis *displays seasonal variation and increases in winter [3-6]. Because breathing cold air (e.g., -1°C at 10-20 l/min) reduces the nasopharyngeal temperature from 34°C at room temperature to ~26°C within several minutes and for extended periods of time [7], the human nasopharyngeal flora is repeatedly exposed to rapid downshifts of environmental temperature. In addition to viral infections that pave the way for subsequent secondary bacterial infections [8], the rapid downshift of temperature induces adaptive events in the residential upper respiratory tract flora that may lead to the transition from asymptomatic colonization to bacterial secondary infection. Our previous findings established that a 26°C cold shock upregulates the expression of UspA1, a major adhesin and putative virulence factor of *M. catarrhalis*, and promotes *M. catarrhalis *adherence to upper respiratory tract cells via enhanced binding to fibronectin [9,10]. Exposure of *M. catarrhalis *to 26°C also increases the outer membrane protein (OMP)-mediated release of the proinflammatory cytokine IL-8 in pharyngeal epithelial cells and reduces the expression of porin M35, which may affect the resistance to aminopenicillins [10,11].

Among the various putative virulence factors that have been identified to date, several other proteinaceous antigens including lactoferrin-binding proteins (LbpA/B), transferrin-binding proteins (TbpA/B), CopB, UspA2 and Hemagglutinin (Hag/MID) may be involved in the cold shock response and thus be important in adapting to and colonizing the human host. Iron is an essential nutrient for most bacteria and *M. catarrhalis *overcomes the host's restriction of free iron through the evolution of iron acquisition systems which enable it to use lactoferrin, transferrin, hemoglobin, and hemin as iron sources. The primary site of *M. catarrhalis *entry into the human host is the nasopharynx, where lactoferrin is the predominant source of iron. Therefore, efficient iron acquisition from lactoferrin is an important virulence factor for pathogenic bacteria. The surface protein CopB is involved in the ability of M.catarrhalis to acquire iron from human transferrin and lactoferrin and is also an important factor responsible for complement evasion [12,13]. UspA2, a major OMP of *M. catarrhalis*, binds vitronectin, a component of both plasma and the extracellular matrix, and confers serum resistance of *M. catarrhalis *[14]. Furthermore, the UspA2 is able to bind human C3 and C4bp protecting *M. catarrhalis *from complement-mediated killing [15,16]. The surface protein Hag/MID that acts as an adhesin and hemagglutinin, exhibits unique immunoglobulin (Ig) D-binding properties and binds to both soluble and membrane-bound IgD on B cells [17-19]. Our previous study demonstrated that exposure of *M. catarrhalis *to 26°C down-regulates *hag *mRNA expression [9], indicating a possible involvement of Hag in the cold shock response.

In the present study we investigated the effect of a 26°C cold shock on the expression of genes involved in iron acquisition, serum resistance and immune evasion. Cold shock induced the expression of genes involved in transferrin/lactoferrin acquisition and enhanced binding of these proteins on the surface of *M. catarrhalis*. Exposure of *M. catarrhalis *to 26°C upregulated the expression of UspA2, a major OMP involved in serum resistance, leading to the improved vitronectin binding. In contrast, cold shock decreased the expression of Hag, a major adhesin mediating B cell response, and reduced IgD-binding on the surface of *M. catarrhalis*.

## Methods

### Bacterial strains and culture conditions

*M*. *catarrhalis *strain O35E, its isogenic *tbpB *(O35E.*tbpB*), *uspA1 *(O35E.uspA1), *uspA2 *(O35E.uspA2), *hag *(O35E.*hag*) and *lpxA *(O35E.*lpxA*) mutants, and clinical isolates 300 and 415 have been described elsewhere [9,20,21]. Bacteria were cultured at 37°C and 200 rpm in brain heart infusion (BHI) broth (Difco) or on BHI agar plates in an atmosphere containing 5% CO_2_. Cold shock experiments were performed as described [9]. Bacteria were grown overnight at 37°C, resuspended in fresh medium and grown to mid-logarithmic phase (optical density at 600 nm [OD_600_] of 0.3). Subsequently, bacteria were exposed to 26°C or 37°C, respectively, for 3 hours (unless otherwise stated). The growth rates of *M. catarrhalis *under iron depletion conditions were evaluated by culturing the bacteria in BHI containing 30 μM desferioxamine (Desferal; Novartis).

### RNA methods

RNA for mRNA expression analysis was isolated and used for complementary DNA (cDNA) synthesis as described elsewhere [9]. Generated cDNA was amplified by semi-quantitative polymerase chain reaction (PCR) using primers for *lbpB *(5'-GCAAGGCGGTAGGGCAGAT-3', 5'-CCTGCTTTTTCGGCGGTGTC-3'), *lbpA *(5'-AACAACGCATTCACAGCACCGATT-3', 5'-GATACCAAGACGAGCGGTGATG-3'), *tbpB *(5'-CAAGCAGGCCGGTGGTATGG-3', 5'-GGTAAATGGGGTGAATGTGGTTGC-3'), *tbpA *(5'-AAGGCGGAGGCAACAGATAAGACA-3', 5'-AGAGCCAGATAATGCCCCAGAGC-3') and 16S ribosomal RNA [rRNA] (5'-AAGGTTTGATC(AC)TGG(CT)TCAG-3', 5'-CTTTACGCCCA(AG)T(AG)A(AT)TCCG-3').

Quantitative real-time PCR was performed in triplicate for both target and normalizer (16S rRNA) genes. No-template controls and RT-negative controls were included in each run. Primers and probes were purchased from Applied Biosystems. Primers for *tbpB *were 5'-TCCTTTCACTTCGCTAAATCGGTTT-3', 5'-CCACACAAGATGCGGTCAAATATAAA-3', and TaqMan probe was 5'-(FAM)CCTTTGTTGGCAACATC-3'. Primers for *uspA2 *were 5'-GCCTTAGACACCAAAGTCAATGC-3', 5'-AAGCTGCCCTAAGTGGTCTATTC-3', and TaqMan probe was 5'-(FAM)TGAAAACGGTATGGCTG-3'. Primers and probes for *hag *and 16S rRNA were used as described elsewhere [9,22]. Relative quantification of gene expression was performed using the comparative threshold method. The ratios obtained after normalization were expressed as folds of change compared with samples isolated from bacteria exposed for 1 h at 37°C.

### Immunoblotting

OM vesicles, composed of OMPs and lipooligosaccharide (LOS), from strain O35E exposed for 3 h to either 26°C or 37°C were prepared by the EDTA buffer method [23]. Samples were resolved by SDS-PAGE using a 7.5% polyacrylamide gel and transferred to polyvinylidene difluoride membranes (Millipore). Lactoferrin binding was detected using mouse anti-human lactoferrin monoclonal antibody (AbD Serotec) and horseradish peroxidase (HRP)-conjugated goat anti-mouse antibody (Sigma). IgA-binding was detected using human saliva samples as the primary antibody source and HRP-conjugated goat anti-human IgA (Sigma) as secondary antibody. Sampling of saliva from healthy volunteers was approved by the local ethics committee.

### Solid-phase lactoferrin binding assay

Detection of lactoferrin binding to *M. catarrhalis *was performed as described elsewhere [24]. Equal amounts of strain O35E grown at 26°C or 37°C for 3 h were spotted onto the nitrocellulose membranes. The blots were blocked in Tris-buffered saline (50 mM Tris buffer containing 0.1 M NaCl [pH 7.0]) containing 0.5% nonfat dry milk and incubated with human lactoferrin (10 μg/mL), followed by a mouse anti-human lactoferrin antibody and developed by using horseradish peroxidase-conjugated goat anti-mouse antibody.

### Two-dimensional gel electrophoresis (2-DE)

**Figure 1:**
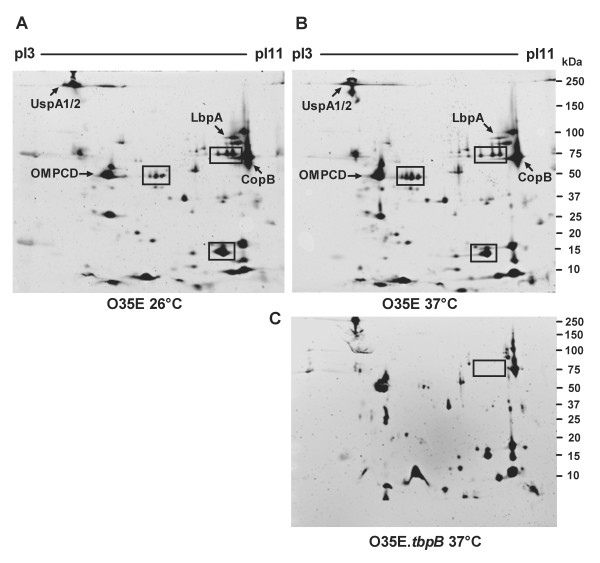
**OM proteome analysis following cold shock in *M. catarrhalis***. OMPs were extracted from a culture of *M. catarrhalis *strain O35E, which was exposed to a 3-hour cold shock at 26°C (A) or to continuous growth at 37°C (B). A collection of 6 gels (3 of each temperature) resulting from three independent experiments was analyzed by ImageMaster^® ^2D Platinum software (Amersham). Three OMPs that are differentially regulated in response to a 26°C cold shock, are indicated in the boxes (A and B). Gel of OMPs isolated from a *M. catarrhalis *O35E.*tbpB *mutant grown at 37°C is shown (C). Identified proteins are labeled. The pI and mass (kDa) values are shown at the top and the right side of each gel.

Analysis of OMPs spots of strain O35E was performed as described previously [25]. To identify the proteins indicated in Figure 1, the MALDI-TOF was used [25]. Protein concentration was determined using the 2-D Quant-Kit (Amersham). Differential analysis was performed using the ImageMaster 2D Platinum software version 5.0 (Amersham) for spot detection, quantification, matching and comparative analysis. The expression level was determined by the relative volume of each spot in the gel and expressed as %Volume (%Vol = (spot volume/Σvolumes of all spots resolved in the gel)). This normalized spot volume takes into account variations due to protein loading and staining by considering the total volume over all the spots present in the gel. A collection of 6 gels (3 of each temperature) resulting from three independent experiments was analyzed. Spots detected by the program were matched between the three gel pairs. Variations in abundance were calculated as the ratio of average values of %Vol between two temperatures. Only spots with a %Vol variation ratio greater than 2 (with significance set at 2-fold change) in the ImageMaster 2D Platinum report were considered relevant.

### Treatment of ***M. catarrhalis ***with lactoferrin

Treatment of *M. catarrhalis *with lactoferrin was performed as described elsewhere [26]. Strain O35E was grown to an OD_600 _of 0.5, resuspended in assay solution containing 0.1% gelatine to a concentration of 10^5 ^CFU/mL prior to the addition of lactoferrin (1 mg/mL, Sigma). Samples were incubated at 37°C for 1 and 3 h followed by plating on BHI agar to determine viability.

### Flow cytometry

Bacteria were exposed to 26°C or 37°C for 3 h. The OD_600 _was adjusted to 0.2, the 200-μL aliquots were washed in PBS-1% BSA, and incubated with 1 μg/mL of lactoferrin or with 1 μg of vitronectin (Millipore) for 1 h. To assess the ability of *M. catarrhalis *to bind salivary lactoferrin, bacteria were preincubated with saliva samples (1:20 dilution) from healthy adults. Bacteria were incubated with mouse anti-human lactoferrin monoclonal antibody (AbD Serotec) or mouse anti-human vitronectin monoclonal antibody (Quidel) followed by incubation with Alexa 488-conjugated goat anti-mouse antibody (Invitrogen) and analyzed on a FACScan cytometer using CellQuest software (version 4.2; BD Bioscience). Anti-human lactoferrin or vitronectin antibodies and Alexa 488-conjugated anti-mouse antibody were added separately as negative controls.

Binding of transferrin to *M. catarrhalis *was analyzed using fluorescein isothiocyanate (FITC)-conjugated human transferrin (0.1 μg/mL, Jackson Immunoresearch).

The ability of *M. catarrhalis *to bind human IgD was analyzed as described elsewhere [27]. Strain O35E, Hag-deficient mutant (O35E.*hag*), LOS-deficient mutant (O35E.*lpxA*) and clinical isolate 300 were exposed to 26°C or 37°C for 3 h, harvested, and incubated with 50% of pooled normal human serum (NHS) as a source of IgD, followed by a FITC-conjugated rabbit anti-human IgD polyclonal antibody (Dako).

The expression of UspA1/A2 and CopB was analyzed using the *uspA1*/*A2*-specific 17C7 and the *copB*-specific 10F3 (1:20) mouse monoclonal antibodies.

### Statistical analysis

Data were expressed as mean ± 1 standard deviation (SD). Differences between groups were analyzed by one-way analysis of variance with a Bonferroni posttest using Prism software (version 5.01; GraphPad). *P*< 0.05 was defined as statistically significant.

## Results

### OM proteome analysis following cold shock in *M. catarrhalis*

To assess cold shock-induced changes in the OM proteome of *M. catarrhalis*, 2-DE analysis was used. OMPs were isolated from a culture of *M. catarrhalis *strain O35E, which was exposed to a 3-hour cold shock at 26°C or to continuous growth at 37°C. A collection of 6 gels (3 of each temperature) resulting from three independent experiments was analyzed. Three OMPs (~75 kDa, pI9; 50 kDa, pI7; and 14 kDa, pI8) were found to be differentially (a greater than twofold change) regulated in response to a 26°C cold shock (Figure 1). Among these proteins, two spots (75 and 15 kDa) were upregulated and one spot (50 kDa) was down-regulated at 26°C (Figure 1A) in comparison with exposure to 37°C (Figure 1B). The 75 kDa spot, which is upregulated at 26°C, was identified by comparing spot pattern of *M. catarrhalis *O35E wild-type and O35E.*tbpB *mutant strain as TbpB (Figure 1C), a peripheral OM lipoprotein possessing transferrin-binding properties, indicating that cold shock may increase iron acquisition, which is important for both growth and virulence.

### Increased expression of genes involved in iron acquisition of *M. catarrhalis *induced by cold shock

**Figure 2:**
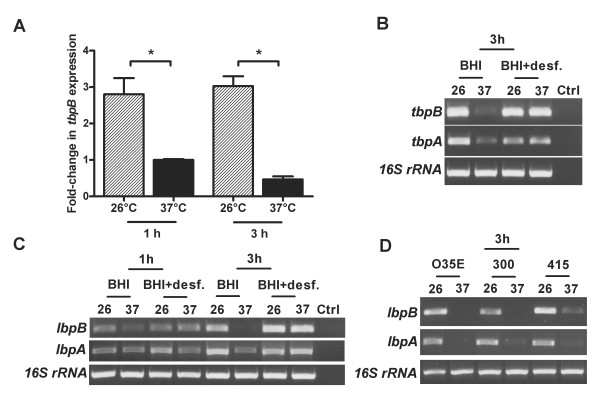
**Increased expression of genes involved in iron acquisition of *M. catarrhalis *due to cold shock**. *A*, increased mRNA levels of *M. catarrhalis tbpB *following to cold shock. Strain O35E, grown to midlogarithmic phase, was exposed for 1 h and 3 h to 26°C or 37°C. RNA was analyzed by quantitative real-time reverse-transcription PCR to determine the amount of *tbpB *and 16S rRNA transcripts. The graph shows one of three representative experiments done in triplicate. Data are presented as means ± 1 standard deviation. *, *P*< 0.05 for 26°C versus 37°C (one-way analysis of variance). *B*, *C *and *D*, increased mRNA levels of *M. catarrhalis tbpB*, *tbpA*, *lbpB *and *lbpA *due to cold shock. *M. catarrhalis *strain O35E and clinical isolates 300 and 415, grown to midlogarithmic phase either in BHI medium or in medium containing 30 μM desferioxamine (Desferal^®^), were exposed to 26°C or 37°C. RNA was analyzed by semi-quantitative reverse-transcription PCR. PCR products were analyzed on 1.5% agarose gels, stained with ethidium bromide and subsequently visualized. To confirm equal loading, PCR for 16S rRNA was performed in parallel. Ctrl indicates control reactions with no cDNA templates.

To confirm the contribution of TbpB in the cold shock response, we assessed the *tbpB *mRNA expression level of strain O35E exposed to either 26°C or 37°C. The expression level of *tbpB *was significantly increased at 26°C in comparison to expression at 37°C (Figure 2A). A similar expression pattern of *tbpB *was also observed in *M. catarrhalis *clinical isolate 300 (data not shown). Cold shock at 26°C also enhanced the mRNA level of *tbpA*, an integral OM transferrin binding protein (Figure 2B). Low free iron conditions (30 μM of desferioxamine in the medium) caused an increase in gene transcription in bacteria grown at 37°C to a level similar to that seen in cells exposed to cold shock.

Because lactoferrin rather than transferrin is the primary carrier of iron on mucosal surfaces and lactoferrin binding proteins are thought to be important virulence factors in some gram-negative bacteria [28], we investigated whether cold shock affects the expression of these genes. As shown in Figure 2, cold shock increased the mRNA level of *lbpB *and *lbpA *genes in strain O35E after 3 h of incubation at 26°C (Figure 2C). Furthermore, cold shock increased the transcriptional level of *lbpA *and *lbpB *of other clinical isolates indicating that this effect is a general characteristic of *M. catarrhalis *(Figure 2D).

### Enhanced binding of transferrin and lactoferrin on the surface of *M. catarrhalis *induced by cold shock

**Figure 3:**
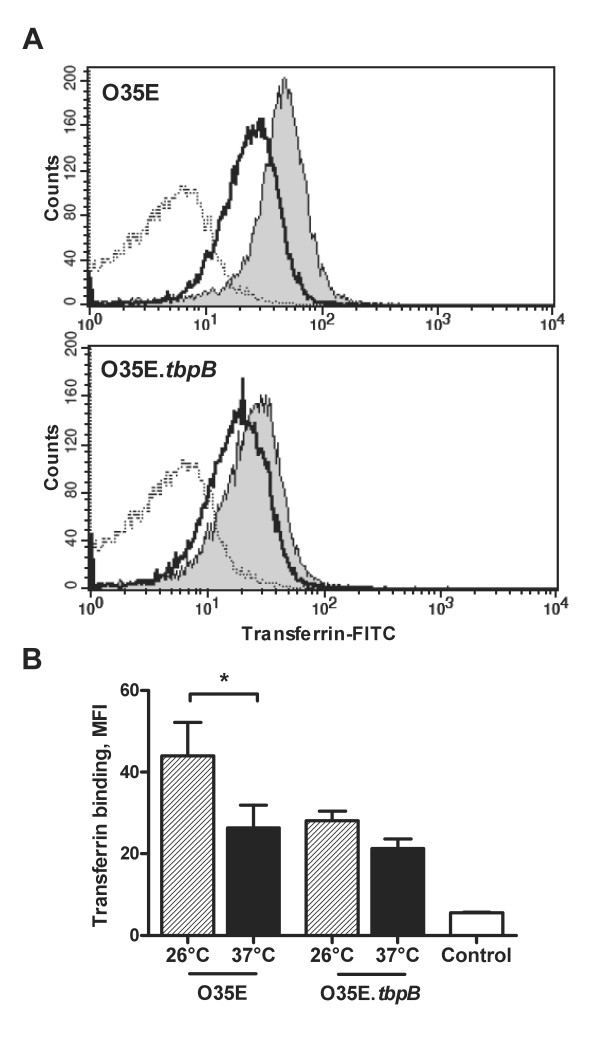
**Increase in the binding of transferrin on the surface of *M. catarrhalis *as a result of cold shock**. *A*, strain O35E and its isogenic mutant O35E.*tbpB *exposed to 26°C or 37°C for 3 h were incubated with fluorescein isothiocyanate (FITC)-conjugated transferrin (0.1 μg/mL) and flow cytometry analysis was performed. Shown are representative flow cytometry profiles of strain O35E and O35E.*tbpB *after exposure at 26°C (*gray*) or at 37°C (*black*), which demonstrate that TbpB is required for maximum binding of transferrin on the surface of cold shock-induced *Moraxella catarrhalis*. The dotted line represents the negative control (bacteria only). The mean fluorescence intensity ± 1 standard deviation for three experiments performed is shown in panel *B*. *, *P*< 0.05 for 26°C versus 37°C (one-way analysis of variance).

Because a temperature drop from 37°C to 26°C induces an increase in the copy numbers of genes involved in iron acquisition, we investigated whether it also affects the binding to human transferrin and lactoferrin. Strain O35E and its TbpB-deficient mutant were exposed to 26°C or 37°C and evaluated for their ability to bind transferrin. Binding to transferrin was increased when bacteria were exposed to 26°C (Figure 3A and 3B). The absence of TbpB reduced binding to transferrin, indicating that TbpB is required for maximum binding of transferrin on the surface of cold shock-induced *M. catarrhalis*.

**Figure 4:**
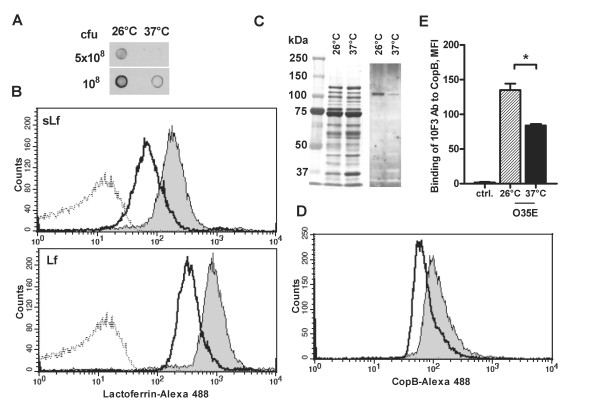
**Increase in the binding of lactoferrin on the surface of *M. catarrhalis *as a result of cold shock**. *A*, solid-phase lactoferrin binding assay. *B*, strain O35E exposed to 26°C or to 37°C for 3 h was preincubated with saliva samples from healthy adults or human milk lactoferrin, followed by a mouse anti-human lactoferrin antibody. Alexa 488-conjugated anti-mouse antibody was added, followed by flow-cytometric analysis. Representative flow-cytometric profiles of *M. catarrhalis *strain O35E after exposure at 26°C (*gray*) or at 37°C (*black*) show cold shock-dependent binding to salivary lactoferrin (sLf) and lactoferrin isolated from human milk (Lf). The dotted line represents the negative control (bacteria incubated with secondary antibodies only). *C*, binding of human lactoferrin to OMPs isolated from *M. catarrhalis *strain O35E exposed to 26°C or 37°C was analyzed by SDS-PAGE Coomassie blue staining (left panel) and Western blot (right panel). Proteins were probed with human lactoferrin. Molecular weight markers in kDa are indicated to the left. *D*, increase in CopB surface expression due to cold shock. Strain O35E exposed to 26°C or to 37°C for 3 h was incubated with the *copB*-specific 10F3 mouse monoclonal antibodies, followed by Alexa 488-conjugated anti-mouse antibody. Representative flow-cytometric profiles of *M. catarrhalis *strain O35E after exposure at 26°C (*gray*) or at 37°C (*black*) show cold shock-dependent CopB upregulation. The mean fluorescence intensity ± 1 standard deviation for 2 experiments performed is shown (E). *, p < 0.05 for 26°C versus 37°C (one-way analysis of variance).

Binding to lactoferrin in a whole-cell solid-phase binding assay was significantly increased when bacteria were exposed to 26°C, in comparison with exposure to 37°C (Figure 4A). The surface binding of human salivary and milk lactoferrin (sLf and Lf, respectively) was further quantitated using flow cytometry, resulting in a clear shift of fluorescence intensity for *M. catarrhalis *exposed at 26°C (Figure 4B). Immunoblot analysis revealed that OMPs isolated from strain O35E exposed to 26°C display a greater lactoferrin binding capacity in comparison to OMPs isolated from cells exposed to 37°C (Figure 4C). The band with a strong lactoferrin-binding capacity and an apparent molecular weight of 100 kDa most likely represents LbpA because only LbpA (103 kDa), an integral OMP, is able to bind lactoferrin and is essential for iron acquisition from lactoferrin, whereas LbpB only plays a facilitating role [24].

Since lactoferrin is an antibacterial protein found in human secretions [26], it was important to determine its bactericidal effect on *M. catarrhalis*. No bactericidal effect was observed when *M. catarrhalis *strain O35E was incubated with human lactoferrin (data not shown).

Because CopB is involved in the ability of M.catarrhalis to acquire iron from human lactoferrin and transferrin, we assessed the expression of this protein following cold shock. Flow cytometry analysis demonstrates that exposure of M.catarrhalis strain O35E to 26°C increases the expression of CopB on the bacterial surface (Figure 4D and 4E).

### Cold shock results in upregulation of UspA2 and increases the binding of vitronectin on the surface of *M. catarrhalis*

**Figure 5:**
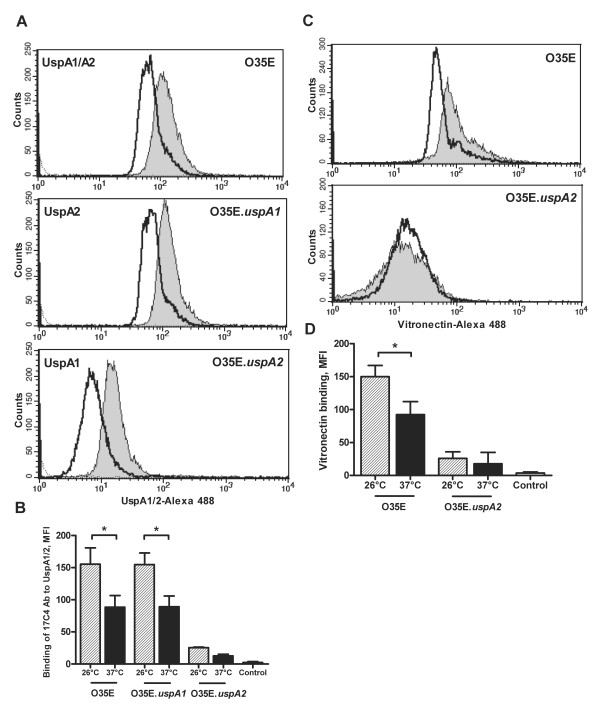
**Cold shock results in upregulation of UspA2 and increases the binding of vitronectin on the surface of *M. catarrhalis***. Representative flow-cytometric profiles of *M. catarrhalis *strains O35E, O35E.us*pA1 *and O35E.us*pA2 *after exposure at 26°C (*gray*) or at 37°C (*black*) show cold shock-dependent UspA1/A2 upregulation (A) and UspA2-dependent binding to vitronectin (C). The dotted line represents the negative control (bacteria incubated with secondary antibodies only). The mean fluorescence intensity ± 1 standard deviation for 2 experiments performed is shown (B and D). *, p < 0.05 for 26°C versus 37°C (one-way analysis of variance).

To investigate the involvement of UspA2 in the cold shock response, we assessed *uspA2 *mRNA expression levels after exposure of *M. catarrhalis *to 26°C or 37°C. Quantitative RT-PCR showed no significant differences between 26°C and 37°C with respect to the relative amount of *uspA2 *mRNA (data not shown). We determined the expression of UspA2 after cold shock on the surface of *M. catarrhalis*. Because the monoclonal antibody 17C7 recognizes both UspA1/A2, we used UspA1 and UspA2 mutants, respectively, of strain O35E. Expression of both UspA1 and UspA2 were increased on the surface of *M. catarrhalis *after cold shock (Figure 5A and 5B). UspA2 mediates serum resistance of *M. catarrhalis *by binding vitronectin. Given that cold shock induces UspA2 expression, we hypothesized that a temperature downshift might increase surface binding of vitronectin. We preincubated *M. catarrhalis *grown at 26°C or 37°C with human vitronectin and determined vitronectin binding by flow cytomertry. Binding to vitronectin was increased when bacteria were exposed to 26°C (Figure 5C and 5D). The absence of UspA2 diminished binding of vitronectin but did not abolish it, possibly due to UspA1 interactions with vitronectin [29]. Serum bactericidal assay with *M. catarrhalis *strain O35E exposed to 26°C or 37°C demonstrated that cold shock did not influence serum resistance of O35E strain (data not shown).

### Cold shock influences *hag *expression and binding of human IgD on the surface of *M. catarrhalis*

**Figure 6:**
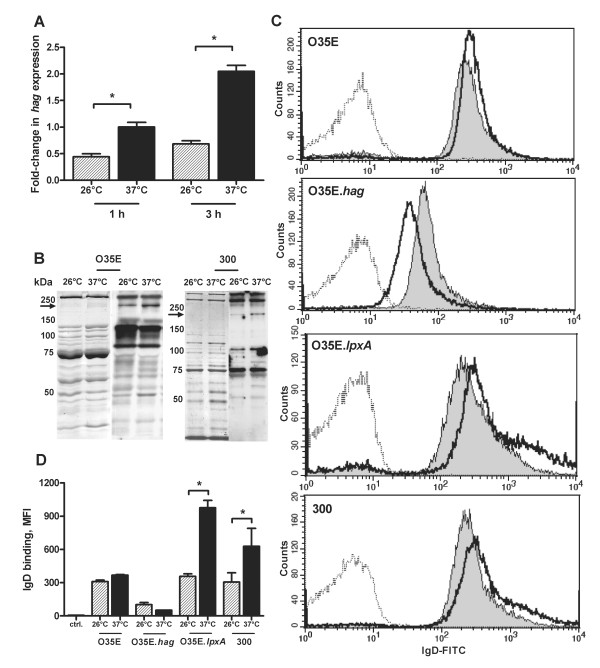
**Cold shock influences *hag *expression and binding of human IgD on the surface of *M. catarrhalis***. *A*, expression level of *hag *mRNA. Strain O35E grown to midlogarithmic phase, was exposed for 1 h or 3 h to 26°C or 37°C. RNA was analyzed by quantitative reverse-transcription PCR to determine the amount of *hag *and 16S rRNA transcripts. The graph shows one of three representative experiments done in triplicate. Data are presented as means ± 1 standard deviation. *B*, expression of Hag following cold shock. The corresponding OMPs profiles of *M. catarrhalis *strains O35E and 300 were visualized by Coomassie brilliant blue staining (left panel) and Western blot analysis (right panel) after SDS-PAGE. Proteins were probed with saliva samples. The arrow indicates the position of Hag (approximately 200 kDa). Molecular weight markers in kDa are indicated to the left. *C*, binding of *M. catarrhalis *to IgD. Representative flow cytometry profiles of *M. catarrhalis *strain O35E, Hag-deficient mutant (O35E.*hag*), LOS-deficient mutant (O35E.*lpxA*) and clinical isolate 300 after exposure at 26°C (*gray*) or 37°C (*black*) show Hag-dependent binding to IgD. The dotted line represents the negative control (bacteria incubated with secondary antibodies only). The mean fluorescence intensity ± 1 standard deviation for 2 experiments performed is shown (D). *, p < 0.05 for 26°C versus 37°C (one-way analysis of variance).

To investigate the contribution of Hag to the cold shock response, we assessed the *hag *mRNA expression level of strain O35E exposed to either 26°C or 37 C. The expression level of *hag *was significantly reduced at 26°C in comparison to expression at 37°C (Figure 6A). Addressing the question whether a decreased mRNA copy number of *hag *at 26°C translates into decreased expression of Hag on the bacterial surface, we performed immunoblot analysis with OMPs preparations of strains O35E and 300 exposed at 26°C or 37°C for 3 h using human salivary IgA antibodies which specifically recognize surface exposed OMPs, including Hag [20]. Immunoblot analysis revealed that *M. catarrhalis *strains O35E and 300 exposed at 26°C expressed smaller amounts of Hag protein compared to bacteria incubated at 37°C (Figure 6B). The Hag-deficient O35E.*hag *strain did not bind the Hag-specific salivary IgA (data not shown). Since Hag has been found to be responsible for *M. catarrhalis *binding to IgD, we investigated IgD-binding on the surface of bacteria grown at 26°C or 37°C. Flow cytometric analysis demonstrated a slight decrease of IgD-binding on the surface of strain O35E after cold shock, while IgD-binding to clinical isolate 300 was clearly reduced at 26°C (Figure 6C and 6D). The Hag-deficient mutant displayed an overall reduced IgD-binding level with increased binding of IgD at 26°C in comparison to 37°C, suggesting that other OM components might antagonize the Hag-mediated IgD-binding following cold shock. This concept is supported by previous findings demonstrating the ability of mucosal IgD to recognize lipopolysaccaride, a key cell wall component of gram-negative bacteria [30]. Indeed, the LOS-deficient mutant of *M. catarrhalis *strain O35E exhibited significantly decreased binding of IgD on the surface of cold shock-induced bacteria in comparison with exposure to 37°C (Figure 6C and 6D).

## Discussion

In this study, we have analyzed the cold shock-induced changes in the OM proteome of *M. catarrhalis *and identified TbpB, whose expression was increased more than two-fold after a 26°C cold shock, as a member of the iron acquisition systems that is important for both growth and virulence. Our data demonstrate that the expression of transferrin receptors and transferrin binding on the bacterial surface were also increased when *M. catarrhalis *was exposed to a 26°C cold shock. Transferrin is predominantly found in serum and in serous exudates. During pronounced inflammation, it is likely that the local tissue damage results in the transsudation of various iron sources, including transferrin, to mucosal surfaces acting as additional iron sources for *M. catarrhalis *[31].

We also demonstrate that a 26°C cold shock increased the expression of genes involved in lactoferrin acquisition and enhanced lactoferrin binding on the surface of *M. catarrhalis*. The overall presence of lactoferrin receptors in *M. catarrhalis *isolates suggests its important role in colonization or infection [32]. In our previous study we demonstrated that exposure of *M. catarrhalis *to 26°C increases the release of proinflammatory cytokine IL-8 in pharyngeal epithelial cells likely leading to the increased inflammation [10]. Thus, greater local concentrations of IL-8 would promote enhanced recruitment and influx of neutrophils that release lactoferrin from their secondary granules, which contribute to lactoferrin levels both locally and in the circulation [33,34]. On the other hand, increased expression of *M. catarrhalis *lactoferrin binding proteins following cold shock would facilitate the binding and acquisition of iron from lactoferrin to support growth of bacteria in the mucosal environment. It has been shown that supplemental lactoferrin can enhance the virulence of meningococcal infection in mice [35]. In addition to iron acquisition, lactoferrin receptors may provide protection against anti-bacterial cationic peptides (eg, lactoferricin) and reduce complement-mediated killing. The pneumococcal surface protein PspA binds lactoferrin and protects *Streptococcus pneumoniae *against the antibacterial effect of lactoferricin [26]. The release of LbpB from the cell surface by a NalP protease protects *Neisseria meningitidis *against bactericidal antibodies [36]. Therefore, increased expression of lactoferrin receptors and enhanced binding of lactoferrin on the surface of bacteria following cold shock might be associated with enhanced protection of *M. catarrhalis *against anti-bacterial cationic peptides and bactericidal antibodies.

The level of UspA2 protein that afforded serum resistance in the bactericidal activity assay has been shown to correlate with increased binding of vitronectin [37]. Our results indicate that cold shock upregulates the UspA2 protein expression and promotes *M. catarrhalis *binding to vitronectin. Increased UspA2 protein expression at 26°C was not the result of higher copy number of *uspA2 *mRNA, indicating that post-transciptional mechanisms are involved in upregulation of this protein after cold shock [38]. Cold shock did not influence the serum resistance of O35E strain indicating that *M. catarrhalis *strains may need to maintain a certain threshold level of UspA2 protein necessary to evade host defenses. Most seroresistant *M. catarrhalis *strains express at 37°C sufficient levels of UspA2 to mediate serum resistance [37]. It is conceivable that cold shock would increase UspA2 expression and vitronectin binding in *M. catarrhalis *strains constitutively expressing low levels of UspA2, leading to the enhanced serum resistance.

The infant population during the first year of life possesses a substantial proportion of IgD in saliva [39]. The interaction between Hag and IgD mediates B cell endocytosis and killing of *M*. *catarrhalis *whereas non-IgD-binding bacteria were not taken up by B cells [27]. Furthermore, IgD-stimulated mucosal basophils release antimicrobial factors inhibiting the replication of *M. catarrhalis *[30]. Here we demonstrate that cold shock at 26°C reduces the mRNA expression level of *hag*, Hag protein expression and the Hag-mediated binding of human IgD to the surface of *M. catarrhalis*. Decreased copy numbers of *hag *at 26°C were also found in other clinical isolates indicating that this effect is a general characteristic of seroresistant *M. catarrhalis *[9]. Therefore, reduced expression of Hag and decreased binding of IgD on the bacterial surface following cold shock might lead to reduced stimulation of B cells and increased survival by prevention of endocytosis by these cells as well as to decreased stimulation of basophils leading to reduced release of antimicrobal factors. However, the presence of specific IgD against LOS triggered increased recognition of bacteria following cold shock (Figure 6). Consequently, children who lack LOS-specific IgD may be more susceptible to *M. catarrhalis *infections, particularly after exposure to cold air.

Three OMPs were found to be differentially (a greater than two fold change) regulated in response to a 26°C cold shock (Figure 1), while immunoblot and flow cytometric analysis revealed that several other OMPs are also involved in cold shock response. The lack of some differentially regulated OMPs in the 2-DE pattern might be the result of difficult identification or low abundance. Furthermore, protein spots with a fold change below the indicated threshold were considered by the Image Master 2D program as not relevant.

Thus, cold shock, which occurs when humans breathe cold air [7], is a physiologic phenomenon during the cold season and entails a range of adaptive events in the residential upper respiratory tract flora that lead to the stimulation of nutrient (e.g., iron)-acquistion, serum resistance and immune evasion potentially resulting in increased bacterial density on the nasopharyngeal surface. Clinical studies in children have demonstrated that the density of *M. catarrhalis *in the nasopharynx is positively associated with prolonged respiratory tract symptoms and a greater likelihood of purulent otitis media [40,41]. This study demonstrates that a 26°C cold shock induces the expression of genes involved in transferrin and lactoferrin acquisition, and enhances binding of these proteins on the surface of *M. catarrhalis*. Exposure of *M. catarrhalis *to 26°C upregulates both CopB and UspA2 expression, the latter leading to improved vitronectin binding on the surface of bacteria. In contrast, cold shock decreases the expression of Hag and reduces the IgD-binding on the surface of *M. catarrhalis*. These findings indicate that cold air in the human upper respiratory tract induces in *M. catarrhalis *a complex of adaptive mechanisms that may enable the bacterium to target their host cellular receptors or soluble effectors and consequently to display enhanced growth, colonization and virulence.

## Conclusions

A physiologic cold shock as it occurs when humans breathe cold air for prolonged periods of time increases the capacity of *M. catarrhalis *for iron uptake from human lactoferrin and transferrin, enhances the capacity of *M. catarrhalis *to bind vitronectin, which neutralizes the lethal effect of human complement, and decreases IgD-binding by hemagglutinin. These data support the notion that *M. catarrhalis *uses physiologic exposure to cold air to upregulate pivotal survival systems in the human pharynx that may contribute to bacterial virulence. Thus, cold shock may exert adaptive events in at least one member of the residential upper respiratory tract flora of facultative pathogens, which may increase the bacterial density on the respiratory tract mucosal surface (which in turn is associated with an increased likelihood of acute otitis media).

## Authors' contributions

VS conceived of the study, designed the experiments, conducted the majority of the experimental work and wrote the manuscript. RT performed the comparative SDS-PAGE analyses. AS performed and analyzed the 2-DE and MALDI-TOF experiments. CA conceived the study, designed the experiments and finalized the manuscript. All authors read and approved the final manuscript.
